# Using Essential Oils to Reduce *Yersinia enterocolitica* in Minced Meat and in Biofilms

**DOI:** 10.3390/foods13050806

**Published:** 2024-03-06

**Authors:** Suzana Vidaković Knežević, Slobodan Knežević, Jelena Vranešević, Dubravka Milanov, Zoran Ružić, Nedjeljko Karabasil, Sunčica Kocić-Tanackov

**Affiliations:** 1 Scientific Veterinary Institute “Novi Sad”, 21000 Novi Sad, Serbia; suzana@niv.ns.ac.rs (S.V.K.); slobodan.knezevic@niv.ns.ac.rs (S.K.); jelenababic@niv.ns.ac.rs (J.V.); dubravka@niv.ns.ac.rs (D.M.); 2 Department of Veterinary Medicine, Faculty of Agriculture, University of Novi Sad, 21000 Novi Sad, Serbia; ruzicvet@gmail.com; 3 Faculty of Veterinary Medicine, University of Belgrade, 11000 Belgrade, Serbia; nedja@vet.bg.ac.rs; 4 Faculty of Technology Novi Sad, University of Novi Sad, 21000 Novi Sad, Serbia

**Keywords:** *Yersinia enterocolitica*, cinnamon essential oil, oregano essential oil, thyme essential oil, anti-*Yersinia* activity, minced pork meat, biofilm, antibiofilm activity

## Abstract

Yersiniosis, one of the leading foodborne infections in the European Union, is caused by *Yersinia enterocolitica.* In this study, the antibacterial and antibiofilm effects of cinnamon (*Cinnamomum zeylanicum* Nees), clove (*Syzygium aromaticum* L.), oregano (*Origanum vulgare* L.), rosemary (*Rosmarinus officinalis* L.), thyme (*Thymus vulgaris* L.), and winter savory (*Satureja montana* L.) essential oils were investigated against *Y. enterocolitica* strains belonging to the bioserotype 4/O:3. Cinnamon essential oil showed the highest antibacterial activity, with an MIC value 0.09 µL/mL, followed by oregano and thyme essential oils, with MIC values from 0.09 to 0.18 µL/mL, and from 0.18 to 0.23 µL/mL, respectively. Thyme essential oil at 0.23 µL/g (MIC) and at 0.46 µL/g (2MIC) significantly (*p* < 0.05) reduced the number of *Y. enterocolitica* by 0.38 log CFU/g and 0.64 log CFU/g, respectively, in minced pork meat during storage at 4 °C for 4 days. The *Y. enterocolitica* strains formed biofilms at 15 °C and 37 °C in tryptic soy broth and Luria–Bertani broth, while no biofilms were obtained at 5 °C, and in meat broth nutrient media. Applying the minimum bactericidal concentrations of cinnamon, clove, oregano, rosemary, thyme, and winter savory essential oils on preformed biofilms led to significant reductions being observed in the range from 45.34% to 78.89%. A scanning electron microscopy assay showed the devastating impact of oregano and thyme essential oils on the morphology of *Y. enterocolitica* bacterial cells. In conclusion, the results of this study show that essential oils possess high anti-*Yersinia* and antibiofilm effects.

## 1. Introduction

Currently, the genus *Yersinia* consists of 28 species (*Y. aldovae*, *Y. aleksiciae*, *Y. alsatica*, *Y. artesiana*, *Y. bercovieri*, *Y. canariae*, *Y. enterocolitica*, *Y. entomophaga*, *Y. frederiksenii*, *Y. hibernica*, *Y. intermedia*, *Y. kristensenii*, *Y. massiliensis*, *Y. mollaretii*, *Y. nurmii*, *Y. occitanica*, *Y. pekkanenii*, *Y. pestis*, *Y. philomiragia*, *Y. proxima*, *Y. rochesterensis*, *Y. pseudotuberculosis*, *Y. rohdei*, *Y. ruckeri*, *Y. similis*, *Y. thracica*, *Y. vastinensis,* and *Y. wautersii*) [1]. Three species of *Yersinia* are human pathogens, including *Y. pestis*, the causative agent of plague, and *Y. enterocolitica* and *Y. pseudotuberculosis*, the enteropathogen species [2,3]. *Y. enterocolitica* is divided into six biotypes, and into over 70 serotypes [4,5]. According to pathogenicity, biotype 1A is non-pathogenic, biotypes 2, 3, 4, and 5 are low-pathogenic, while the biotype 1B is highly pathogenic [3,6]. This facultative anaerobic, Gram-negative bacterium may cause human infections with various symptoms, including fever, abdominal pain, diarrhea, ileitis, pseudoappendicitis, mesenteric lymphadenitis, arthritis, septicemia, and mortality [7,8].

*Y. enterocolitica*, the subject of our study, is transmitted to humans as a foodborne or waterborne pathogen [4,5]. Although yersiniosis occurs after the consumption of various contaminated food of both animal and plant origin, contaminated pork is the main source of *Y. enterocolitica* in European countries [5]. Pigs are considered the main asymptomatic reservoir of *Y. enterocolitica*, especially bioserotype 4/O:3 [9]. The prevalence in Europe is estimated to be up to 93% [10]. The prevalence of *Y. enterocolitica* in pigs varies worldwide [11,12], and depends on several factors including the farm management system, conditions in slaughterhouses, and the detection methodology [13]. During slaughter, *Y. enterocolitica* from tonsils and the intestine can contaminate carcasses and work surfaces at slaughterhouses. This can lead to the cross-contamination of the meat. A low number of *Y. enterocolitica* in minced meat is characterized as a high microbiological risk factor for consumers [14], because *Y. enterocolitica* can survive and replicate at refrigerated temperatures [9].

One of the survival strategies of bacterial pathogens is the ability to form biofilms [15]. In the food industry, biofilms are mainly responsible for microbial contamination. Biofilms are complex three-dimensional communities of surface-attached bacteria protected by an extracellular matrix. Here, bacterial cells communicate through a signaling mechanism known as quorum sensing (QS) [15]. *Y. enterocolitica* are able to form biofilms through five stages of activating the QS system [16]. In the first stage, bacterial cells adhere to a surface. Next, extracellular polymeric substances (EPSs) are made, and bacterial cells become irreversibly attached. The EPSs form a complex organic polymer matrix which consists of polysaccharides, proteins, lipids, nucleic acid, and other substances [17]. In the third stage, the biofilm architecture is formed, and the biofilm becomes mature. The maximum bacterial cell density is reached at the next stage. After that, the biofilm releases bacterial cells that can attach and contaminate new surfaces and form new biofilms [15]. Insufficient and ineffective sanitation allows the biofilm to persist on food-contacting surfaces, presenting a constant source of microbial contamination of food. Contaminated food may cause foodborne diseases, which are, along with antimicrobial resistance, a global public health problem [18]. Further, biofilms can damage equipment and cause food spoilage all leading to increased costs in the food sector [16,19].

Although *Y. enterocolitica* infection is mainly asymptomatic, antibiotics are often used as a conventional method of treatment. The World Health Organization suggests the use of chloramphenicol, gentamicin, cotrimoxazole, tetracyclines, fluoroquinolones, and third-generation cephalosporins. Yet, antibiotic resistance has been observed [20]. Because of this, it is important to find an adequate treatment strategy against *Y. enterocolitica* biofilms, and against the *Y. enterocolitica* present on meat.

One of the measures against *Y. enterocolitica* may be natural volatile liquids, named as essential oils (EOs). EOs are complex mixtures of secondary metabolites originating from aromatic and/or medicinal plants with wide biological activities [21]. They are obtained from different parts of aromatic plants (oregano, rosemary, thyme, sage, lavender, basil, and many others) [22,23], including roots, leaves, flowers, fruits, seeds, by extraction methods, of which hydrodistillation is the most common [24]. A wide range of EO biological activities have been thoroughly examined, including antimicrobial, antiviral, antifungal, anticancer, antioxidant, and anti-inflammatory activity [24,25]. The corresponding mechanisms of action are many, and they have been previously reported [24]. Different EOs show strong antibacterial activity against many foodborne pathogens, including *Salmonella* Enteritidis, *Salmonella* Typhimurium, *Listeria monocytogenes*, *Campylobacter jejuni*, *Camplylobacter coli*, *Escherichia coli*, *Staphylococcus aureus*, *Bacillus subtilis*, *Bacillus cereus*, and others [21]. The chemical composition of EOs is responsible for their antimicrobial properties [25]. According to Durofil et al. [20], there are some promising data about EOs’ activity against *Y. enterocolitica*. In the last two decades, extensive investigations have involved EOs of *Ocimum basilicum*, *Origanum vulgare*, *Rosmarinus officinalis*, and *Thymus vulgaris*. Articles have shown several minimum inhibitory concentration (MIC) values of the *Cinnamomum zeylanicum* (17.5 µL/L, 75 µg/mL), *Syzygium aromaticum* (8.7 µL/L), *Origanum vulgare* (75 µg/mL, 20 µL/mL, 2.5 µL/mL, 0.6 mg/mL, 4.4 µL/L), *Rosmarinus officinalis* (0.075 mg/mL, 20 µL/mL, 8 µg/mL), *Thymus vulgaris* (1.2 mg/mL, 32 µg/mL, 34.9 µL/L, <0.2 µL/mL), and *Satureja montana* (0.32%) EOs against *Y. enterocolitica*, all depending on their origin, plant part, chemical composition, and performed assay [20]. However, in addition to *in vitro* studies, EOs deserve further research in terms of their effect on *Y. enterocolitica*.

To the best of our knowledge, no research has examined the reduction in *Y. enterocolitica* biofilms by cinnamon (*Cinnamomum zeylanicum* Nees), clove (*Syzygium aromaticum* L.), oregano (*Origanum vulgare* L.), rosemary (*Rosmarinus officinalis* L.), thyme (*Thymus vulgaris* L.), and winter savory (*Satureja montana* L.). The novelty of this research lies in the different approach of measuring the antimicrobial and antibiofilm activity of six commercially available EOs against *Y. enterocolitica* 4/O:3. Withal, all *Y. enterocolitica* strains included in this research were isolated from a slaughterhouse located in Serbia. Additionally, the winter savory EO was obtained from organic plants cultivated in Southeast Serbia, making it a locally available option for the *Y. enterocolitica* control. The aims of this research were (I) to determine the antibacterial activity of selected EOs against *Y. enterocolitica* in minced pork meat, (II) to determine the ability of *Y. enterocolitica* strains to form biofilms in different nutrient media and temperature conditions, and (III) to investigate the influence of the selected EOs on the formed *Y. enterocolitica* biofilms.

## 2. Materials and Methods

### 2.1. Essential Oils

Essential oils of cinnamon (*Cinnamomum zeylanicum* Nees, Sri Lanka) (CIEO), clove (*Syzygium aromaticum* L., India) (CLEO), oregano (*Origanum vulgare* L., India) (OREO), rosemary (*Rosmarinus officinalis* L., Spain) (ROEO), thyme (*Thymus vulgaris* L., India) (THEO), and winter savory (*Satureja montana* L., Serbia) (WSEO) were selected for this experiment. All EOs were commercially available from Terra Co, Novi Sad, Serbia, and Siempreviva oils, Niš, Serbia. For identification of EO compounds, a gas chromatograph GC 7890B coupled with an MS 5977A mass spectrometer (Agilent Technologies, Santa Clara, CA, USA) was used, as in the previous study by Vidaković Knežević et al. [26].

### 2.2. Bacterial Strains

Three *Y. enterocolitica* strains (Y4/1, Y9, and Y14) were previously isolated from pig tonsils and belonged to the bioserotype 4/O:3 [27]. Until examination, strains were stored frozen in tryptic soy broth (TSB) (Oxoid, UK) with the addition of 20% glycerol at −80 °C.

### 2.3. Determination of Minimum Inhibitory Concentration (MIC) and Minimum Bactericidal Concentration (MBC)

The EOs were prepared in DMSO (Lach-Ner SRO, Prague, Czech Republic). The broth microdilution method reported by Kocić-Tanackov et al. [28] was used to determine the MICs and MBCs of the EOs. Briefly, 100 µL of EO was mixed with 100 µL of Muller–Hinton broth (Oxoid, Basingstoke, UK) in the first well of a microtiter plate. After that, a 1:1 serial dilution was made, reaching the concentration 0.23 µL/mL. All wells were filled with 10 µL *Y. enterocolitica* suspension (10^8^ CFU/mL). Incubation was maintained at 37 °C for 24 h. Then, the content of each well was inoculated onto Muller–Hinton agar (Biokar Diagnostic, Beauvais, France) and incubated at 37 °C for 24 h. The lowest concentrations of EOs that inhibited the visible growth of *Y. enterocolitica* were defined as MICs, while the lowest concentrations of EOs with no growth after subculturing onto Muller–Hinton agar were defined as MBCs.

### 2.4. Preparation of Minced Meat

The fresh pork *Quadriceps femoris* was minced in a sterile grinder with a No. 4 disc, and portions of 10 g were placed into sterile Petri dishes. The minced pork meat samples were inoculated with ca. 10^4^–10^5^ CFU *Y. enterocolitica* strain Y4/1. Then, the samples were treated with MIC and 2MIC values of OREO (0.09 µL/g and 0.18 µL/g), and THEO (0.23 µL/g and 0.46 µL/g). All samples were homogenized using a sterile glass rod, and stored in Petri dishes for 4 days at 4 ± 1 °C.

### 2.5. Bacterial Enumeration

*Y. enterocolitica* count was performed by adding all 10 g of minced pork meat sample into a stomacher bag containing 90 mL sterilized peptone water (Biokar Diagnostics, Beauvais, France). After homogenization, decimal dilution was performed followed by spread-plating on CIN agar (Yersinia selective agar base CM0653, with Yersinia Selective Supplement SR0109, Oxoid, UK), and incubation at 30 ± 1 °C for 24 h. The suspect colonies, small (≤1 mm), smooth, with a red center and translucent rim, were further examined according to the standard method [29].

### 2.6. Biofilm Formation

The capability of *Y. enterocolitica* strains to produce biofilms in TSB, meat broth (MB) (Oxoid, UK), and Luria–Bertani broth (LB) (Oxoid, UK) at three different temperatures, including 5 °C, 15 °C, and 37 °C for 48 h was tested according to a previously reported protocol [30]. Briefly, *Y. enterocolitica* strains were subcultured in TSB overnight at 37 °C, and then diluted in TSB, MB, and LB at the ratio of 1:40. Aliquots of 200 µL were inoculated into wells of a sterile 96-well microtiter plate with flat bottoms (Sarstedt, Nimbrecht, Germany) and incubated at different temperatures for 48 h. After incubation, the *Y. enterocolitica* cells that did not adhere were discarded, rinsed with physiological saline (3 × 250 μL/well), and air-dried. Following this, fixation (250 μL/well of 96% ethanol for 20 min), staining (250 μL of 0.3% crystal violet (Fluka, Sigma-Aldrich, Germany) for 20 min), rinsing (tapped water), air drying, and recording the optical density at 550 nm (OD_550_) on the ASYS Expert Plus Microtitration Reader (Biochrom, Cambridge, UK) were performed. All *Y. enterocolitica* strains were classified as non-biofilm formers (OD ≤ ODC), weak biofilm formers (ODC ≤ OD ≤ (2 × ODC)), moderate biofilm formers ((2 × ODC) < OD ≤ (4 × ODC)), or strong biofilm formers (OD > (4 × ODC)) [31].

### 2.7. Reduction in Biofilm

The reduction in biofilms by EOs was performed according to a previously reported methodology [30]. Following the procedure from Section 2.6, the biofilms of *Y. enterocolitica* strains were formed. After discarding and washing non-adherent *Y. enterocolitica* cells, the adhered biofilm was treated with an aliquot of 200 µL solution of MBC concentrations of EOs in TSB for 48 h at 15 °C and 37 °C. As in the biofilm formation procedure, after washing, the remained biomass was stained with 0.3% crystal violet. OD_550_ of remaining biofilms were measured, and the reductions were calculated following Equation (1):

Biofilm reduction (%) = [(OD_GROWTH CONTROL_ − OD_SAMPLE_)/OD_GROWTH CONTROL_] × 100, (1)

### 2.8. Scanning Electron Microscopy (SEM) Assay

Suspensions of *Y. enterocolitica* strain Y9 (100 μL) were inoculated on the surfaces of sterile SS 304 stainless steel coupons (10 × 10 × 1 mm) for 3 h at 37 °C, and placed in a 12-well plate. Then, the SS 304 stainless steel coupons were rinsed with 3 mL of physiological saline, treated with 2 mL LB broth (control), MIC of OREO (0.18 µL/mL), and MIC of THEO (0.45 µL/mL), and incubated at 37 °C for 24 h. After incubation, the SS 304 stainless steel coupons were rinsed with physiological saline. The fixation was performed using 4% glutaraldehyde at 5 °C overnight. Once again, the SS 304 stainless steel coupons were rinsed with physiological saline and dehydrated with a series of graded ethanol (30%, 50%, 60%, 70%, 90%, and 96%). The SS 304 stainless steel coupons were air-dried and coated with gold (Bal-Tec SCD005 Sputter Coater, BAL-TEC AG, Balzers, Liechtenstein) prior to SEM analysis (JMS SEM 6460 LV, JEOL Ltd., Tokyo, Japan).

### 2.9. Statistical Analysis

The research findings are presented as mean ± standard deviation (SD). Using statistical software R version 3.2.2 (R Foundation for Statistical Computing, Vienna, Austria), the outcomes were evaluated with an analysis of variance (ANOVA) test, followed by a Duncan’s test. The statistical comparison was evaluated at *p* < 0.05. All assays were performed in triplicate.

## 3. Results and Discussion

### 3.1. Chemical Composition of Essential Oils

The detailed chemical compositions of CIEO, CLEO, OREO, ROEO, THEO, and WSEO have been reported in a previous study [30]. The main component of CIEO was cinnamaldehyde (74.93%), followed by ascabin (9.01%). CLEO and OREO were rich in eugenol (85.14%) and carvacrol (81.00%), respectively. The main components of ROEO were α-pinene (28.23%) and borneol (24.87%), followed by α-terpineol (11.86%) and 1,8-cineole (11.54%). THEO was rich in *p*-cymene (40.91%) and thymol (40.36%). The main component of WSEO was carvacrol (50.45%), followed by *p*-cymene (15.73%) and γ-terpinene (11.43%). Usually, EOs contain one to three main components at a high concentration, while the others may be at a significantly lower concentration, even as traces [24]. The chemical composition of EOs varies quantitatively and qualitatively. These differences are the outcome of the affect of endogenous and exogenous factors. Endogenous factors include the anatomical and physiological characteristics of plants, i.e., plant genetics (species, ecotype, chemotype), plant origin, season, vegetative phase and parts of plants, physiological and biochemical pathways, degree of development, and metabolic processes of plants. Exogenous factors include the external environment, i.e., climate and habitat conditions (temperature, humidity, windiness, soil composition, geographical origin), cultivation conditions, i.e., agrotechnical measures (method and time of harvesting) and techniques applied after harvesting (methods of drying, extraction, time distillations, and storage conditions) [25,32].

### 3.2. Antibacterial Effect of Essential Oils

**Figure 1:**
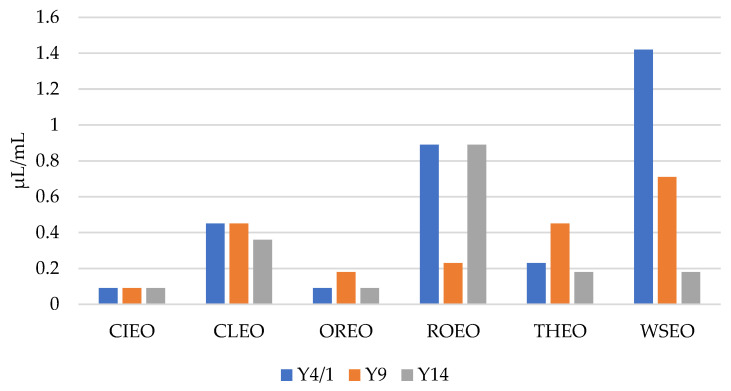
MICs of cinnamon (*Cinnamomum zeylanicum* Nees) (CIEO), clove (*Syzygium aromaticum* L.) (CLEO), oregano (*Origanum vulgare* L.) (OREO), rosemary (*Rosmarinus officinalis* L.) (ROEO), thyme (*Thymus vulgaris* L.) (THEO), and winter savory (*Satureja montana* L.) (WSEO) against the three strains of *Y. enterocolitica*.

**Figure 2:**
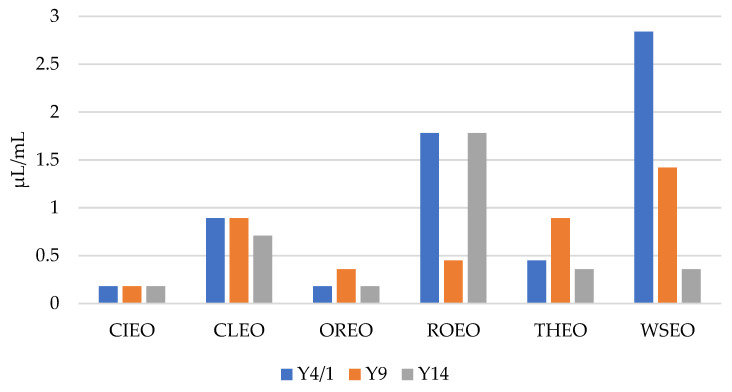
MBCs of cinnamon (*Cinnamomum zeylanicum* Nees) (CIEO), clove (*Syzygium aromaticum* L.) (CLEO), oregano (*Origanum vulgare* L.) (OREO), rosemary (*Rosmarinus officinalis* L.) (ROEO), thyme (*Thymus vulgaris* L.) (THEO), and winter savory (*Satureja montana* L.) (WSEO) against the three strains of *Y. enterocolitica*.

As presented in Figure 1 and Figure 2, the results show that the MICs and MBCs of the EOs for the *Y. enterocolitica* strains ranged from 0.09 µL/mL to 1.42 µL/mL, and 0.18 µL/mL to 2.84 µL/mL, respectively. CIEO manifested the strongest antibacterial activity, with an MIC value of 0.09 µL/mL for all three *Y. enterocolitica* strains, followed by OREO, with an MIC value of 0.09 µL/mL for two *Y. enterocolitica* strains, Y4/1 and Y14. The MIC value for the Y9 strain was 0.18 µL/mL. The widest range of MICs, from 0.18 µL/mL to 1.42 µL/mL, was obtained with WSEO. The antibacterial activity of different EOs, including CIEO, OREO, ROEO, and THEO, against *Y. enterocolitica* have been previously reported [9,20]. The MIC and MBC values of CIEO, CLEO, OREO, ROEO, THEO, and WSEO for *Y. enterocolitica* are similar to the values for *S.* Enteritidis, *S.* Typhimurium, and *L. monocytogenes* obtained using the EOs of same origin [26,30]. According to the review of Durofil et al. [20], which included results from more than 50 researchers, EOs originating from plants that belong to the *Lamiaceae* and *Lauracea* families possess high activity against *Yersinia*. This statement is in accordance with our study, with the fact that oregano, thyme, and winter savory belong to the *Lamiaceae* family, while cinnamon belongs to the *Lauracea* family.

### 3.3. Antibacterial Effect of OREO and THEO on Y. enterocolitica Inoculated in Minced Pork Meat

As reported in Table 1, the initial population of *Y. enterocolitica* was between 4.31 log CFU/g and 4.46 log CFU/g in all the treatments, without a significant difference (*p* > 0.05). The OREO at 0.09 µL/g and 0.18 µL/g did not lower the number of *Y. enterocolitica* in the minced pork meat during the 4 days of storage at 4 ± 1 °C. On the contrary, the THEO significantly (*p* < 0.05) lowered the number of *Y. enterocolitica* in the minced pork meat at 0.23 µL/g and 0.46 µL/g, for 0.38 log CFU/g and 0.64 log CFU/g, respectively. Similar to the outcomes of our research, the addition of OREO in barbecued chicken meat did not reduce the number of *Y. enterocolitica* during 72 h of storage [33]. According to our knowledge, this is the first reported research to examine the efficiency of THEO in minced pork meat against *Y. enterocolitica.* The reduction in *Y. enterocolitica* may be due to the high content of thymol, a phenolic monoterpene able to alter the composition of the fatty acids of the cell membrane, and violate its integrity, causing the leakage of intracellular materials [34]. Oregano and thyme are regularly used as spices in meat products, and can elevate the antibacterial, antioxidant, and sensory properties, and consequently extend the shelf-life [35]. However, the required amounts of both EOs in food models are much higher than those obtained in *in vitro* studies, and can cause negative organoleptic effects [36], with an emphasis on strong aroma. Food components, such as protein and fat, are able to reduce the antibacterial effect of EOs [32]. In addition, *Y. enterocolitica* is able to activate its adaption mechanisms when exposed to oregano EO to protect itself, including a reduction in energy consumption for mobility, flagellum formation, and QS to ensure normal physiological function [37].

### 3.4. Formation of Biofilms by Y. enterocolitica

The production of biofilms by *Y. enterocolitica* strains on the surface of polystyrene wells with the three different nutrient media (TSB, MB, and LB broth) at three different temperatures (5 °C, 15 °C, and 37 °C) over 48 h are shown in Table 2. It is well known that the formation of biofilms is under the influence of the characteristics of bacteria, the surface, and the environment conditions [22]. In this study, at 5 °C, none of *Y. enterocolitica* strains formed a biofilm. The same was noticed with the MB, regardless of the temperature. The *Y. enterocolitica* strains were capable of forming weak and moderate biofilms, while no strong biofilms were observed in this study. The Y4/1 strain formed one moderate and three weak biofilms. The Y9 strain formed only a moderate biofilm under two different conditions, while the Y14 strain formed one moderate and two weak biofilms. Similar findings have been reported previously [38,39,40]. According to Wang et al. [40] and our results, *Y. enterocolitica* can form biofilms under conditions simulating a pork slaughterhouse. Unlike the MB used in our study, the MJ (meat juice) was more suitable for biofilm formation compared to TSB. The biofilm in MJ was more resistant to sanitization treatment, physical washing, and starvation when compared to the biofilm formed in TSB [40].

In our study, only 3 h of adhesion was enough for the *Y. enterocolitica* cells to attach to the surface and start forming a biofilm. Other studies have shown that adhesion can be observed after 6–8 h, while the formation of a mature biofilm takes 48–72 h [41]. The plasmid for *Yersinia* virulence (pYV) is essential for the surface properties of *Y. enterocolitica* [42]. The capability of *Y. enterocolitica* to produce biofilms contributes to its pathogenicity and adaptability [43], which increases the risk of contaminating food-contacting surfaces in processing plants and final products. *Y. enterocolitica* biofilm formations are more common at higher temperatures, so keeping the temperature cool may reduce the bacterial adhesion on food-contacting surfaces and reduce the biofilm formation in production plants.

### 3.5. Biofilm Reduction

Biofilm reduction was conducted on selected moderate biofilm formers. The MBC concentrations of EOs reduced the 48 h old *Y. enterocolitica* biofilms in the range from 45.34% to 78.89%, as shown in Table 3. To reduce established biofilms, EO must penetrate the exopolysaccharide matrix, reach the protected surface-attached bacterial cells, and alter the QS system. The QS system controls biofilm formation, so the strategy in biofilm reduction is targeting QS [16].

The major components of EOs, such as cinnamaldehyde, eugenol, carvacrol, *p*-cymene, and thymol, are responsible for their antibiofilm effects. However, the components of EOs present in smaller amounts play a significant synergistic role [22]. Former studies have suggested that low concentrations of cinnamaldehyde (0.078 mg/mL) can repress the production of *Y. enterocolitica* biofilm [16]. In a previous study, it was shown that extracts from 12 edible plants inhibited the biofilm production of *Y. enterocolitica* [44]. Natural antibacterial agents cause abnormal expression of a few important genes, including *luxS*, *glgC*, *envZ*, *ompF*, *kdpD*, and *cydB*, resulting in the damage of the biofilm [45]. Generally, planktonic cells are more sensitive to EOs compared with cells protected in biofilms. Therefore, the strategy to control biofilms in production plants is an important part of food safety. The antibiofilm mechanisms of EOs are different and are not entirely clear [22], which can also be concluded from the results of this study. Namely, the achieved reduction in *Y. enterocolitica* biofilms varied within the same applied EO. The lowest difference was observed applying ROEO (14.75%), while the highest difference was observed applying THEO (32.8%). Differences in biofilm reduction could be explained by the influence of temperature. Lower reduction rates were observed at 15 °C, compared with 37 °C. The temperature of 30 °C is the optimal growth temperature for *Y. enterocolitica*, so the bacterial cells in the biofilm formed and treated at 37 °C could be stronger and more resistant to the applied EOs. Additionally, EOs could exhibit different affinities for different surfaces. Polystyrene has a hydrophobic surface, and attracts EOs more than hydrophilic stainless steel [22].

### 3.6. SEM

**Figure 3:**
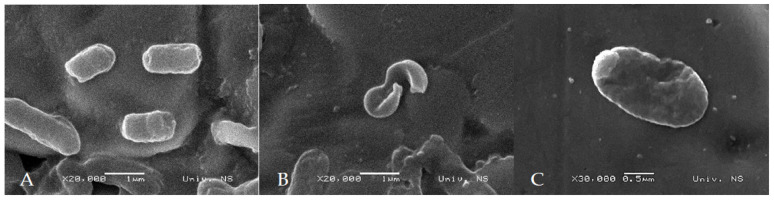
SEM micrographs of *Y. enterocolitica* cells on SS 304 stainless steel coupons: untreated (**A**) and treated with oregano (*Origanum vulgare* L.) essential oil (**B**) and thyme (*Thymus vulgaris* L.) essential oil (**C**). Magnifications: (**A**) ×20,000, (**B**) ×20,000, (**C**) ×30,000.

The SEM assay was performed on the *Y. enterocolitica* strain Y9. This strain was chosen because of its ability to form the strongest biofilms compared with the other two *Y. enterocolitica* strains. Figure 3 shows SEM micrographs of the untreated *Y. enterocolitica* cells, and the *Y. enterocolitica* cells treated with MIC amounts of OREO (0.18 µL/mL), and THEO (0.45 µL/mL). The untreated *Y. enterocolitica* cells have their typical morphological appearance; a smooth surface and rod-shaped structure [45], while the *Y. enterocolitica* cells treated with OREO and THEO showed damage, with deformed shapes, rough surfaces, and membrane rupture. The EO components, such as carvacrol, *p*-cymene, and thymol, act on bacterial cells by various antimicrobial mechanisms, including the attack of the phospholipid bilayer, disrupting enzyme systems, metabolic pathways, and the genetic material of bacterial cells, causing structural and functional damages to the bacterial cell membrane, that eventually lead to cell death [36,46]. Some of the bacteria cells treated with thymol showed swelling [47]. The bacteria cells treated with carvacrol and thymol modified the lipid profile, resulting in an increase in saturated C16 and C18 fatty acids, and a decrease in unsaturated C18 fatty acids, causing membrane structural alterations and permeability [48]. Carvacrol has an effect on the proteins of the outer membrane of Gram-negative bacteria, which allows Gram-negative bacteria to be more resistant to EOs and their compounds compared to Gram-positive bacteria [47].

## 4. Conclusions

The prevalence of *Y. enterocolitica* is high, and as the leading foodborne infection agent, this pathogen deserves more attention. *In vitro* studies have shown that the use of EOs may be an effective treatment against *Y. enterocolitica*. CIEO, CLEO, OREO, ROEO, THEO, and WSEO showed high anti-*Yersinia* effects, with MIC values > 0.09 µL/mL. However, the fact that only the THEO (MIC and 2MIC) was able to reduce the number of *Y. enterocolitica* in minced pork meat during storage at 4 ± 1 °C for 4 days shows the limited practical application of EOs in food at the concentrations obtained in *in vitro* studies. This study also shows the ability of *Y. enterocolitica* to form biofilms in different conditions, regarding temperatures and available nutrients. *Y. enterocolitica* was able to attach to polystyrene and stainless steel surfaces, and started to form biofilms in just 3 h. The CIEO, CLEO, OREO, ROEO, THEO, and WSEO were able to reduce the biofilms of *Y. enterocolitica* strains formed at 15 °C and 37 °C in TSB and LB broth. The applied EOs reduced the biomass of the preformed biofilms by up to 78.89%. SEM showed that OREO and THEO influenced the typical morphological appearance of the *Y. enterocolitica* cells, causing a deformed shape and membrane rupture. This is evidence that environmentally friendly EOs may be used to control foodborne pathogenic biofilms present on food-contacting surfaces made of polystyrene and stainless steel. However, further research is necessary to find adequate EO concentrations applicable to food and surfaces in order to control *Y. enterocolitica*, before the industrial application of EOs.

## Figures and Tables

**Table 1:** Antibacterial effect of oregano (*Origanum vulgare* L.) (OREO) and thyme (*Thymus vulgaris* L.) (THEO) on *Y. enterocolitica* inoculated in minced pork meat (log CFU/g).

Treatments	Days				
	0	1	2	3	4
Control	4.39 ± 0.11 ^aC^	4.55 ± 0.27 ^aC^	5.32 ± 0.13 ^aB^	5.49 ± 0.20 ^aB^	5.84 ± 0.07 ^aA^
OREO 0.09 µL/g	4.41 ± 0.07 ^aD^	4.62 ± 0.25 ^aD^	5.23 ± 0.08 ^aC^	5.50 ± 0.24 ^aB^	5.79 ± 0.23 ^aA^
OREO 0.18 µL/g	4.46 ± 0.14 ^aC^	4.54 ± 0.21 ^aC^	5.40 ± 0.15 ^aB^	5.51 ± 0.23 ^aB^	5.75 ± 0.19 ^aA^
THEO 0.23 µL/g	4.36 ± 0.10 ^aC^	4.60 ± 0.23 ^aB^	5.32 ± 0.12 ^aA^	5.37 ± 0.19 ^aA^	5.46 ± 0.15 ^bA^
THEO 0.46 µL/g	4.31 ± 0.08 ^aD^	4.63 ± 0.25 ^aC^	4.95 ± 0.26 ^bB^	5.07 ± 0.10 ^bAB^	5.20 ± 0.12 ^cA^

Means within a column followed by different small letters (a, b, c) are statistically significant (*p* < 0.05) between the EO concentrations. Means within a row followed by different big letters (A, B, C, D) are statistically significant (*p* < 0.05) between the days of storage.

**Table 2:** Production of biofilm by *Y. enterocolitica* on polystyrene surface under different conditions of nutrient media and temperatures.

Strains	TSB			MB			LB		
	5 °C	15 °C	37 °C	5 °C	15 °C	37 °C	5 °C	15 °C	37 °C
Y4/1	0.102 ± 0.008 °	0.264 ± 0.060 *	0.197 ± 0.089 *	0.092 ± 0.006 °	0.131 ± 0.015 °	0.109 ± 0.024 °	0.016 ± 0.004 °	0.350 ± 0.055 **	0.144 ± 0.043 *
Y9	0.099 ± 0.008 °	0.411 ± 0.090 **	0.382 ± 0.062 **	0.092 ± 0.008 °	0.133 ± 0.008 °	0.104 ± 0.031 °	0.011 ± 0.005 °	0.138 ± 0.012 °	0.115 ± 0.034 °
Y14	0.108 ± 0.010 °	0.305 ± 0.090 *	0.295 ± 0.070 **	0.097 ± 0.009 °	0.163 ± 0.026 °	0.113 ± 0.016 °	0.015 ± 0.006 °	0.254 ± 0.081 *	0.115 ± 0.028 °

Values are mean OD_550_ ± SD. Biofilm classification: °—non-biofilm-former; *—weak biofilm former; **—moderate biofilm former. Cut-off values: TSB (5 °C) = 0.150; MB (5 °C) = 0.161; LB (5 °C) = 0.113; TSB (15 °C) = 0.173; MB (15 °C) = 0.167; LB (15 °C) = 0.159; TSB (37 °C) = 0.108; MB (37 °C) = 0.133; LB (37 °C) = 0.123.

**Table 3:** Reduction (%) in *Y. enterocolitica* biofilms formed at polystyrene surface exposed to cinnamon (*Cinnamomum zeylanicum* Nees) (CIEO), clove (*Syzygium aromaticum* L.) (CLEO), oregano (*Origanum vulgare* L.) (OREO), rosemary (*Rosmarinus officinalis* L.) (ROEO), thyme (*Thymus vulgaris* L.) (THEO), and winter savory (*Satureja montana* L.) (WSEO) essential oils.

Strains	Nutrient Media and Temperature	Essential Oils					
		CIEO	CLEO	OREO	ROEO	THEO	WSEO
Y4/1	LB 15 °C	66.90 ^a^	73.45 ^c^	72.07 ^bc^	71.12 ^bc^	72.17 ^bc^	70.21 ^b^
Y9	TSB 37 °C	62.94 ^bc^	60.65 ^ab^	54.73 ^a^	67.32 ^c^	57.57 ^ab^	59.62 ^ab^
Y9	TSB 15 °C	78.59 ^a^	76.87 ^a^	78.89 ^a^	78.45 ^a^	78.14 ^a^	76.42 ^a^
Y14	TSB 37 °C	62.46 ^b^	63.70 ^b^	70.37 ^b^	63.70 ^b^	45.34 ^a^	62.51 ^b^

Values within a row followed by different letters (a, b, c) are statistically significant (*p* < 0.05) between the effect of EOs.

## Data Availability

The original contributions presented in the study are included in the article, further inquiries can be directed to the corresponding author.
